# The financial and social impacts of the COVID-19 pandemic on youth with eating disorders, their families, clinicians and the mental health system: a mixed methods cost analysis

**DOI:** 10.1186/s40337-024-00986-1

**Published:** 2024-03-29

**Authors:** Nicole Obeid, Patricia Silva-Roy, Linda Booij, Jennifer S. Coelho, Gina Dimitropoulos, Debra K. Katzman

**Affiliations:** 1 Eating Disorders Research Lab, Children’s Hospital of Eastern Ontario Research Institute, 401 Smyth Rd, Ottawa, ON K1H 8L1 Canada; 2 Department of Psychiatry, University of Ottawa, Ottawa, ON Canada; 3 Eating Disorders Continuum, Douglas Mental Health University Institute, Montreal, QC Canada; 4 Department of Psychiatry, McGill University, Montreal, QC Canada; 5 Provincial Specialized Eating Disorders Program for Children and Adolescents, BC Children’s Hospital, Vancouver, BC Canada; 6 Department of Psychiatry, University of British Columbia, Vancouver, BC Canada; 7 Calgary Eating Disorder Program, Alberta Health Services, Calgary, AB Canada; 8 Department of Psychiatry, University of Calgary, Calgary, AB Canada; 9 Division of Adolescent Medicine, Department of Pediatrics, Hospital for Sick Children, Toronto, ON Canada; 10 Department of Pediatrics, University of Toronto, Toronto, ON Canada

**Keywords:** Direct and indirect medical costs, Eating disorders, COVID-19, Mixed-methods

## Abstract

**Background:**

The onset of the COVID-19 pandemic has had an adverse impact on children, youth, and families with eating disorders (EDs). The COVID-19 pandemic exacerbated pre-existing personal and financial costs to youth, caregivers, and health professionals accessing or delivering ED services. The objectives of this mixed methods study were to (1) understand the indirect, direct medical and non-medical costs reported by youth, caregivers, and clinicians; (2) understand how the COVID-19 pandemic may have impacted these costs, and (3) explore implications of these costs with regards to barriers and resources to inform future decisions for the ED system of care.

**Methods:**

Youth (aged 16–25 years) with lived/living experience, primary caregivers, clinicians, and decision-makers were recruited with support from various partners across Canada to complete group specific surveys. A total of 117 participants responded to the survey. From those respondents**,** 21 individuals volunteered to further participate in either a discussion group or individual interview to provide additional insights on costs.

**Results:**

Youth and primary caregivers reported costs relating to private services, transportation and impacts of not attending school or work. Additionally, primary caregivers reported the top direct medical cost being special food or nutritional supplements (82.8%). In discussion groups, youth and caregivers elaborated further on the challenges with long waitlists and cancelled services, impact on siblings and effect on family dynamics. Clinicians and decision-makers reported increased work expectations (64.3%) and fear/isolation due to COVID-19 in the workplace (58.9%). Through discussion groups, clinicians expanded further on the toll these expectations took on their personal life. Approximately 1 in 3 health professionals reported contemplating leaving their position in 1–2 years, with greater than 60% of this group stating this is directly related to working during the pandemic.

**Conclusions:**

Findings demonstrate the need for increased support for youth and caregivers when accessing ED services both during crisis and non-crisis times. Additionally, attention must be given to acknowledging the experience of health professionals to support better retention and resource management as they continue to navigate challenges in the health care system.

**Supplementary Information:**

The online version contains supplementary material available at 10.1186/s40337-024-00986-1.

## Abstract (plain language summary)

The COVID-19 pandemic has had significant impacts for children, youth and families with eating disorders (EDs) and those who care for them. The objective of this study was to understand the impacts on financial and personal costs to youth, caregivers and clinicians when either accessing or delivering ED services during the pandemic. This study consisted of an online survey and discussion groups with youth (16-25 years old) with lived experience of an ED, primary caregivers, clinicians and decision-makers delivering ED services to understand the costs they experienced. Although many reported costs were financial, including direct medical costs (i.e. private therapy) and direct non-medical costs (i.e. transportation, accommodation costs), there were also significant personal costs to individuals and their families. Healthcare professionals also reported extensive challenges with resources and increased work expectations due to the pandemic context. Acknowledging the costs experienced by young people, families and healthcare professionals during the pandemic allows for the discussion of how we can better support those accessing or delivering ED services in times of crisis and non-crisis.

**Supplementary Information:**

The online version contains supplementary material available at 10.1186/s40337-024-00986-1.

## Introduction

The global COVID-19 pandemic (herein referred to as the pandemic) has had disruptive impacts on many domains of life for children and youth, posing significant impacts to many aspects of their mental health during this unprecedented time [1]. Specifically, the pandemic has had an adverse impact on children, youth, and families with eating disorders (EDs), with a spike in the number and acuity of new and pre-existing EDs in young people compared to prior years [2]. This crisis has resulted in higher rates of hospital admissions and emergency room visits [3–5]. Furthermore, youth in treatment reported increased eating-disordered thoughts and behaviours and decreased motivation for recovery [6–8]. Major elevations in new cases have also been reported, with referrals doubling from previous years [9]. This unprecedented demand, high acuity of cases, and the interruption of services have contributed to increasingly long waitlists [10–13]. Factors such as school and recreational closures, isolation from peers, and increase in social media use have been cited as contributing to these elevations [5, 11], unmasking a global ED public health crisis that affects youth, families, and health professionals alike. 

Research conducted during the early part of the pandemic suggests that primary caregivers of youth with EDs had higher rates of depression and anxiety than caregivers of youth without EDs [14]. Other mixed methods and small-sized qualitative studies of youth and families with EDs during the pandemic have revealed that parents’ financial constraints due to job loss impacted their ability to access or afford care for their children [15]. Changes to daily routines and regulations around attending appointments with their children also contributed to greater overall distress [8, 16]. To date, few quantitative studies have examined the number of different costs and impacts of caregiving of young people with EDs during the pandemic. Without accurate information regarding the implications for families and caregivers, there is a limited ability for carers to support individuals fully and effectively with EDs [17]. Understanding the impacts of the pandemic on families with EDs can inform policy-level strategies to help mitigate the social, personal, and financial costs that may affect caregivers and families during ED treatment.

Health professionals in the field of EDs have also reported significant impacts related to working in the ED field during the pandemic. Concerns include increased workloads without an increase in resources; feelings of uncertainty; lack of support; and needing to shift practices (including the prioritization of medically compromised patients for in-person appointments) whilst rapidly learning new strategies to build a therapeutic alliance virtually [16, 18]. Clinicians working in intensive health care settings (e.g., hospitals) acknowledged similar challenges and a cumulative burden of managing this in addition to their own health and well-being [19]. We are not aware of any studies to date that have examined personal and system level costs associated with being a provider during the pandemic, and specifically an ED provider. Understanding these costs could help put strategies in place to mitigate the burden of the impacts encountered, support the retention of health professionals, and prepare an already strapped workforce for any future shifts in care.

The current study sought to elucidate from diverse community members the different types of costs including the direct and indirect medical, personal, and social costs (and cost-savings, if any) that were experienced in the context of the pandemic as it relates to ED care. This study recruited participants from across Canada, including young people affected by an ED during the pandemic, primary caregivers of youth with an ED, clinicians, and decision-makers working in pediatric and youth (up to 25 years old) ED settings. Using an explanatory sequential mixed method design, we assessed the various costs and impacts that affected individuals during the pandemic by first collecting the quantitative data and then qualitative data. The qualitative data were employed to extend our understanding and interpretation of the quantitative data. The ability to better understand and quantify these costs has implications for policy and decision-makers to better prepare and be adequately resourced for when major shifts in healthcare needs occur and allows for the identification of potential cost savings or ways to mitigate financial, personal, or social costs.

## Methods

### Participants

The present mixed methods study included a survey component followed by discussion groups and semi-structured interviews. Young people with lived/living ED experience, primary caregivers, clinicians, and decision-makers from across Canada were invited to participate in both the survey and discussion group components of the study. Inclusion criteria for all respondents included being a resident in Canada at the time of the study and having proficiency in English or French. Youth respondents were invited to participate if they were between the ages of 16–25 years in order to invite responses from youth who were old enough to encounter different types of costs personally. Primary caregivers were identified as a person who, over the past 12 months, spent the most time helping an individual with an ED and/or disordered eating, consistent with Statistics Canada’s definition of caregivers [19]. Clinicians were defined as a health professional who is trained to work with children or youth (up to 25 years old) with EDs and/or disordered eating. Decision-makers encompassed professionals employed in a health-related or community-based organization in the field of EDs for children or youth who identified as: leads or managers in hospital/intensive or community ED programs, policymakers with a portfolio in EDs or anyone with responsibility overseeing ED services and decisions during the pandemic.

### Procedures

Participants were recruited via the extended networks of the ED national and grant partners involved in the project (n = 43). This included social media channels, sharing in newsletters, emailing network members, and by posting on websites. No incentive was offered for survey completion. The survey was available for a duration of 3 months between December 2022 and March 2023, and was offered in the two official languages of Canada (English and French). Following completion of the survey, participants were invited to participate in a discussion group or interview. Some discussion group participants were also recruited through ED advisory groups. The Research Ethics Board of record for this study was approved where the research was funded (Children's Hospital of Eastern Ontario (CHEO), Ottawa, Canada). Additional approval was obtained by the Research Ethics Board through University of British Columbia /Children’s and Women’s Health Centre of BC and The Hospital for Sick Children. Informed consent for the use of the data in this study was sought as part of the recruitment process.

### Measures

#### Survey

The study team created distinct self-report surveys for the different groups, focusing on identifying and understanding the changes in service delivery and costs incurred as a result of or associated with the pandemic. Survey data were collected and managed using RedCap (Research Electronic Data Capture) [20, 21]. The survey included both multiple-choice and open-ended questions. The surveys were co-developed by the study authors, supported by emerging literature on the impacts of the pandemic on EDs across Canada and other previous costs-of-illness studies [22–24]. The project team engaged with members of the Alberta Youth and Family Councils on EDs to review and refine the youth and primary caregiver surveys that were drafted by the study team. The study assessed a variety of costs, including: (a) direct medical costs, such as medications or therapy, (b) direct non-medical costs, defined as expenditures as a result of the illness but not involved in the direct purchasing of medical services, for example transportation or accommodations, and (c) indirect costs which were defined as the loss of earnings and productivity by the youth or caregiver related to the illness, for example being unable to attend school or work. The surveys had varying number of questions to best capture these costs (Youth: 20-item survey, Caregivers: 22-item survey, Clinicians: 8-item survey, Decision-makers: 10-item survey). Surveys can be found in Additional file 1 (See Additional file 1: Appendix B).

#### Demographic survey

Upon completion of the survey, all respondents completed an additional 10-item demographic survey regarding age, postal code, gender identity, sexual orientation, Indigeneity, race, and disability. Demographic questions were adapted versions of Statistics Canada questions created by the University of Toronto’s Employment Survey which were further adapted by the Canadian Institute for Health Information’s Guidance on the use of Data Collection and Health. Five members of CHEO’s Indigenous Circle and Office of Equity were also engaged to finalize the wording of the questions. Both the cost and demographic self-report surveys were anonymous.

#### Discussion groups

Discussion groups and semi-structured interviews were conducted with youth, caregivers, and clinicians. No decision-makers registered to participate in the discussion groups. Participants completed a written consent form on RedCap prior to discussion. Each discussion group (n = 5) was held with participants from the same stakeholder group (youth, primary caregiver, clinician, or decision-maker). Six participants participated in an individual interview due to scheduling conflicts and personal preference. Discussion groups and interviews were conducted over the course of approximately two months and were facilitated by one or two members of the study team. Discussion groups and interviews included questions about participants’ experiences with the pandemic as it related to accessing or delivering ED services, associated costs, and impacts on their personal wellbeing and family. Participants were also shown a summary of survey responses from their respective group and given the opportunity to reflect on the findings. Discussion groups and interviews were approximately 60 min in length and were conducted virtually via Zoom. All discussion groups and interviews were recorded, transcribed verbatim and de-identified by an employee of the institution who is not a member of the study team. Participants received a $30 gift card following their participation. Discussion group/interview guide can be found in supplementary materials (See Additional file 1: Appendix C).

#### Analysis plan

Descriptive and frequency statistics were conducted to understand group specific trends. Data were analyzed separately for each group, where possible. Qualitative data obtained from the discussion groups and interviews were transcribed verbatim and coded using reflexive thematic analysis [25] using NVivo 14 pro software. A combined deductive-inductive approach was used in coding: a deductive approach was used to generate initial codes from the survey data, including open-text survey responses; inductive codes were generated for data that did not align with this set of codes. Two coders independently started with data familiarisation including casual note taking and reading of the transcripts which followed by systematic coding [26, 27]. Following initial coding, the two coders met to review preliminary codes. The two coders continued coding the rest of the data and continued meeting regularly to generate and refine themes. Although coding was conducted separately for each group, both coders reviewed codes, similarities and differences between groups to expand understanding and develop overall themes across all groups. Coding was an iterative process and disagreements were resolved through discussion to generate deeper interpretations of the data [26].

#### Positionality

As part of reflexive qualitative analysis, the positionality of the two coders is important. Reflexive thematic analysis was appropriate given the deductive lens and existing perspective from the survey prior to analyzing and interpreting the data. With regards to qualitative data analysis, the first coder (PSR) was a research coordinator who conducted most of the discussion groups and survey analysis, therefore had additional understanding of the context. The second coder (SD) was a research assistant who was novel to the study and had not been a part of the survey analysis or discussion group facilitation. SD was provided information on the study and anticipated themes prior to coding. In addition, study author (GD) provided guidance of theme development, using an iterative process and critical realist approach to generate themes. Comparisons across different groups were made using survey and qualitative data where possible.

## Results

In total, 117 individuals completed the survey. Of all respondents, 112 (95.7%) completed the survey in English while 5 (4.3%) completed it in French. Amongst the different participants, 29 (24.7%) responses were from youth with lived/living experience, 29 (24.7%) were from caregivers, 52 (44.4%) were from clinicians, and 4 were (3.4%) from decision makers; 3 (2.6%) responses did not identify which group they were representing. Given the low response rate for the survey among decision-makers, results were combined with clinicians and reported together as health professionals when describing this groups survey results. Respondents came from provinces across Canada: 13 respondents (11.1%) were from British Columbia, 35 (29.9%) from Alberta, 1 (0.9%) from Manitoba, 2 (1.7%) from Saskatchewan, 40 (34.2%) from Ontario, 10 (8.5%) from Quebec, 11 (9.4%), from Atlantic provinces, 0 (0%) from the Territories, and 5 (4.3%) who did not specify their province. The majority of those who responded to the survey identified themselves as women (n = 99, 84.6%) and white (n = 100; 85.5%). Further demographic details of the respondents by group are described in Table 1. High level survey results can be found in Table 2 while more detailed survey results of youth and primary caregivers (Additional file 1: Table 2.1) and health professionals (Additional file 1: Table 2.2) are available in the Supplementary Materials. A total of 48 (41%) survey respondents registered to take part in the discussion groups following the survey. Of those, 21 (44%) individuals went on to participate in the discussion groups/interviews; 5 were youth with lived/living experience of an ED, 10 were caregivers, and 6 were clinicians. Five discussion groups with groups ranging from 2 to 5 participants and 6 individual interviews were held. About 86% of discussion group participants (n = 18) completed the optional pre-discussion group demographic survey which was the same 10-item questionnaire from the survey. This was requested again since the original survey was anonymous and could not be linked to participating discussion group members. Discussion group participants were primarily from Alberta (n = 10) and Ontario (n = 5), with one participant from New Brunswick and one from Quebec. The integrated results of the quantitative and qualitative analysis, including quotes from participants can be found in Table 2. This table was developed through simultaneous comparisons of the data to achieve a deeper understanding of the results.

**Table 1:** Reported demographics of survey respondents

	Youth	Primary caregivers	Health professionals
	n = 29	n = 29	n = 56^a^
*Gender identity (n, %)*^*b*^			
Woman	23 (79.3)	26 (89.7)	48 (85.7)
Man	2 (6.9)	2 (6.9)	8 (14.3)
Non-binary	2 (6.9)	0 (0.0)	0 (0.0)
Other	1 (6.9)	0 (0.0)	2 (3.4)
Age in years (SD)	21.90 (4.61)	46.61 (5.27)	41.44 (11.28)
*Time working in field of EDs, n (%)*			
≤ 6 months	–	–	2 (3.6)
> 6 months but ≤ 12 months	–	–	3(5.5)
> One year but ≤ five years	–	–	13 (2.4)
> 5 years but ≤ 10 years	–	–	15(27.3)
> 10 years	–	–	22(40.0)
*ED diagnosis, n (%)*^*c*^			
Anorexia nervosa	20 (69.0)	24 (82.8)	–
Bulimia nervosa	3 (10.3)	2 (6.9)	–
Binge eating disorder	2 (6.9)	0 (0.0)	–
Avoidant/restrictive food intake disorder	2 (6.9)	1 (3.4)	–
Other specified feeding or eating disorder (OSFED)	7 (24.1)	2 (6.9)	–
Unspecified feeding or eating disorder (UFED)	0 (0.0)	1(3.4)	–
Not had a formal diagnosis	3 (10.3)	0 (0.0)	–

^a^Health professionals include 52 clinicians and 4 decision makers

^b^Respondents were able to select more than one gender identity

^c^Respondents were able to select more than one ED diagnosis for themselves or their loved one

**Table 2:** Side by side survey and discussion group/interview data by stakeholder group and theme

Youth	Survey (See Additional file 1: Table 2.1 for additional details)	Discussion groups/interviews	Discussion group/interview demonstrative quotes
Undergoing and delivering ED treatment led to financial costs	Direct Costs: therapist/psychologist/psychiatrist services (58.6%), medication (44.8%), dietitian (37.9%) Direct non-medical costs: transportation (72.4%), costs with virtual care (44.8%), support for other family members (31.0%) Cost savings: did not experience any cost savings (55.2%)	Cost of private services, medication, transportation, accommodations, and other resources Challenges with virtual services	“My parents ended up having to take money out of my RESP [Registered Education Savings Plan] to pay for some of my therapy” (Participant 5) [Regarding virtual services] “there was the parking savings and maybe a little bit of the gas…the cost was still more for me to not be in person than it would have been to just pay for those things and be there” (Participant 4) “I had a therapist within the eating disorder program … and she had to leave and that really led to a downhill spiral, which ended up leading to like, because my parents couldn’t afford a therapist in the community so it kind of led to a downhill spiral, which led to like longer term care which led to more expenses” (Participant 6)
The pandemic exacerbated personal, family and work-related stressors	Indirect costs: unable to attend school (55.2%), increased feelings of isolation due to COVID-19 (48.3%), unable to work resulting in decreased work productivity (37.9%)	Isolation because of ED symptoms and COVID-19 restrictions Impact of ED on other family members, specifically siblings	“Eating disorder itself is isolating and on top of it COVID” (Participant 17) “I was kind of just very isolated, because I wasn’t working anymore, I wasn’t in school, so I guess I didn’t really know what to do with myself. I think that was really really tough, because I’ve always been like a very bubbly kind of person. Like I’m very social, so I found that really hard to be fully isolated. Even before the pandemic. Like I think I was isolated from the beginning of treatment I would say.” (Participant 12) “I know that my sister spent a lot of time on her own, kind of like waiting, and like she really suffered from that and struggled with her own mental health” (Participant 5)
Challenges with resources for ED treatment	Barriers for services/treatment: long wait lists (58.6%), lack of ED specific treatment (58.6%), cost of services/treatment (51.7%)	Difficulty accessing services (eligibility criteria, geographic barriers to access) Long waitlists Frustration with delayed or cancelled treatment options because of the pandemic	“You had to be like at a certain degree of un-wellness in order to be considered for treatment” (Participant 3) “All these things are being put on pause, but like my eating disorder is not being put on pause and it just kind of left us just like waiting” (Participant 5) “You can’t measure sick. Like sick is sick” (Participant 6) “The only place I’d go would be to treatment, and since it was in the local hospital there was a high risk of contracting the virus. I was scared, but at the time, anywhere was better than home” (Survey response open text)
Systemic impacts of delivering or undergoing ED treatment during the pandemic	Not applicable	Harm from medical professionals’ lack of knowledge in ED care Changes in treatment options due to pandemic	“They weren’t accepting new patients because of the COVID risks and so I was just kind of waiting in the general medical unit but like it was, it felt very much like they were all kind of learning with me … I felt I was giving like little crash courses in eating disorder care versus them telling me. So, I think they’ve learned a lot but came a bit at the cost of my own care.” (Participant 5) “I think one of the biggest barriers not pandemic-wise would have been myself” (” (Participant 12)

Primary caregivers	Survey (See Additional file 1: Table 2.1 for additional details)	Discussion groups/interviews	Discussion group/interview demonstrative quotes
Undergoing and delivering ED treatment led to financial costs	Direct Costs: special food or nutritional supplements (82.8%), medication (62.1%), therapist/psychologist/psychiatrist services (58.6%) Direct non-medical costs: transportation (82.8%), supports for themselves as a primary caregiver (58.6%), support for other family members (37.9%) Cost savings: did not experience any cost savings (41.4%)	Cost of private services, food and nutritional supplements, medication, alternative therapies Insufficient and challenges with eligibility of private insurance/benefit coverage Cost savings with virtual care	“The cost of food has gone up and when you have an eating disorder, you tend to want particular types of food, so that can be a challenge too sometimes” (Participant 8) “It [private service] was very expensive. It was not something that we would have been able to access” (Participant 13) “[Private insurance] covered each one like two-and-a-half sessions” (Participant 17) “Even with benefits, it’s nothing” (Participant 21) “Her [psychotherapist] credentials don’t qualify under my benefit plan… even though she’s got 30 years of experience with eating disorders, that’s not good enough to qualify for benefit coverage”(Participant 16) “[Virtual services] was actually really good for us, and it saved us a whole bunch of money” (Participant 10)
The pandemic exacerbated personal, family and work-related stressors	Indirect costs: unable to work resulting in decreased work productivity (69.0%), increased feelings of isolation due to COVID-19 policies (58.6%)	Personal demands and impact of being a caregiver Impact on other children and spousal relationships Isolation with COVID-19 and isolation of ED	“For a lot of years I had work getting in the way” and that “[The pandemic] did provide me with this year where I was able to be home (Participant 11) “I need to go to therapy now, to deal with all the extra trauma and nobody is paying for that, again” (Participant 21) “My son became like the forgotten child. He was the one who ended up trying to be responsible for my daughter, because we were so, trying to keep her alive, he just had kind of had to fend for himself, and that’s awful” (Participant 7) “Because of COVID we both couldn’t visit on site, so she [other child] was often left at home alone” (Participant 8) “it caused relationship issues between my husband and I” (Participant 7)
Challenges with resources for ED treatment	Barriers for services/treatment: long wait lists (72.4%), lack of access to qualified mental health professionals (69.0%), lack of ED specific treatment (62.1%)	Difficulty accessing services (challenges with primary care, long waitlists, lack of services) Caregiver burden to become expert with limited resources available	“We had a huge waitlist before we ended up in the ER in life threatening stages and then the doors opened” (Participant 20) “You have to learn it yourself. Even when the services are technically available. There is no possible way you can get through it without learning” (Participant 11)
Systemic impacts of delivering or undergoing ED treatment during the pandemic	Not applicable	Challenges with ED treatment (autonomy, lack of communication, lack of continuum of care)	“You can’t ask a young person with an eating disorder what they think about it or what they want. I know that’s coming from a good place. You want to empower adolescents, but you are empowering the eating disorder when you do that” (Participant 11)

Clinicians	Survey (See Additional file 1: Table 2.2 for additional details)^a^	Discussion groups/Interviews	Discussion Group/Interview demonstrative quotes
The pandemic exacerbated personal, family and work-related stressors	Personal costs/impacts: increased work expectations/demands (64.3%), Fear/isolation due to COVID-19 exposure at workplace (58.9%)	Isolation of working during pandemic (relationships with others and family), fear of COVID-19, moral distress, burnout Impact of uncertainty Negative work life balance Appeal of private practice	“I feel like work-life balance definitely has shifted. Before COVID I found it a lot easier.” (Participant 14) “I think there were lots of times during the pandemic where I did a bad job of taking care of myself and therefore I think was less present at work, was less present with my family and that was difficult and I think that in trying to balance my work, my relationship and my relationship with my kids, it always felt like something was out of whack’ (Participant 2)
Challenges with resources for ED treatment	Not applicable	Lack of resources (staff, space, experienced staff, services) Disruptions to treatment capacity and availability	“Very experienced clinicians who go and leave to work in private practice because that’s the only [way] that they can afford to live… and so I think we lose a lot of knowledge” (Participant 2) “At one point we were turning away 70% of the referrals we got” (Participant 1) “Mental health patients again were being discriminated against and that, you know, basically kids were being discharged because we had to make space for acute respiratory beds” (Participant 19)
Systemic impacts of delivering or undergoing ED treatment during the pandemic	Change in work: provide virtual care/services (69.6%), remote work/working from home (51.8%) Personal Costs: technology expenses working remotely (41.1%) Cost Savings: One-time pandemic bonus pay (37.5%)	Managing high rates of patients, challenges with increased work expectations and demands Benefits and cost savings of virtual care	“Nobody is being forced to work more hours than we’re paid for, but at the same time, we have this idea of like we have a 500-person waiting list and we’ve got to see as many people as we can” (Participant 15) [Virtual services]“allowed us to then support more rural or remote areas in a more real time way” (Participant 19)

^a^Survey results include the responses of decision makers

### Quantitative results

#### Youth with lived/living experience

The top direct medical costs not covered by provincial or territorial health care reported by young people who accessed ED services included services provided by a therapist and/or psychiatrist (58.6%), medication (44.8%) and services provided by a dietitian (37.9%). Transportation (78.4%), virtual care (44.8%), and supports for other family members (31.0%) were reported by youth as the top three direct non-medical costs. Indirect or social costs reported by youth included being unable to attend school (55.2%), feelings of isolation/loneliness (48.3%), and decreased work productivity (37.9%). Youth reported barriers to service during the pandemic including: long waitlists (58.6%), lack of ED specific treatment (58.6%), mental health staff shortages (48.3%), and the cost of private treatments (51.7%). In terms of cost-savings, 10.3% of youth survey respondents shared that the use of virtual care led to some savings.

#### Caregivers

The top direct medical costs reported by caregivers when accessing ED services during the pandemic included cost of special food or nutritional supplements (82.8%), medication (62.1%) and private healthcare services (58.6%). Transportation (82.8%), supports for themselves as a primary caregiver (58.6%), and additional supports for other family members (e.g., therapy, group counselling) (37.9%) were among the top direct non-medical costs reported by caregivers in the survey. The top reported indirect cost for caregivers in the survey was the inability to attend work, resulting in decreased work productivity (69.0%). Caregivers reported barriers to accessing services during the pandemic including long wait lists (72.4%), lack of access to qualified mental health professionals (69.0%) and a lack of ED specific treatment (62.1%).

#### Health professionals

Most survey-responding health professionals reported the top personal impact during the pandemic included an increase in work expectations and demands (64.3%) and fear/isolation due to COVID-19 virus exposure at the workplace (58.9%). Approximately 70% of responding clinicians indicated in the survey that they provided virtual care/services throughout the pandemic. In addition to these impacts are also the future costs that may occur as a cumulative result of the pandemic on health professionals. Potential for future increases in staff turnover were detected, with 34.5% of responding health professionals stating they were considering leaving their current position in the next 1–2 years. Of these health professionals, greater than 60% shared that working during the pandemic impacted this decision.

### Qualitative results

Across discussion groups, the themes regarding costs were organized into the following four themes: (1) undergoing and delivering ED treatment led to financial costs; (2) the pandemic exacerbated personal, family and work-related stressors; (3) challenges with resources for ED treatment, and (4) systemic impacts of delivering or undergoing ED treatment during the pandemic.

### Undergoing and delivering ED treatment led to financial costs

Participants described a range of financial costs associated with the COVID-19 pandemic which differed depending on their role. In the discussion groups, young people and caregivers described private services as costly; for some, this direct cost was so high as to completely impede access to these options. Even when caregivers had private benefit packages, these did not necessarily cover the costs associated with private services. Costs extended beyond direct costs associated with services; caregivers described the high costs for food and food supplements for their loved one, aligning with survey results. In contrary, there were no significant financial costs reported by clinicians associated with work during the pandemic. However, a clinician raised challenges with fundraising efforts, suggesting that there were systemic/organization financial impacts.

### The pandemic exacerbated personal, family and work-related stressors

Within discussion groups, youth specifically shared how both the pandemic and their ED led to isolation. Isolation, uncertainty, and changing policies during the pandemic further aggravated the stressors youth and families were already experiencing while navigating treatment. Primary caregivers described how caring for a child with an ED requires 24 h supervision, and that there was some perceived benefit of shifting to work-at-home to aid with this.

In addition to personal impact, caregivers and two youth described how other children in their families experienced distress. Many caregivers indicated that they were not able to care for their other child(ren) like they would have liked to because they were “*surviving*” (Participant 17). Strict visitor policies that did not allow for sibling visits exacerbated this issue by enforcing a choice between visiting one’s child or staying with the sibling. The strain also led to challenges in parenting decisions and spousal relationships. Caregivers described the cumulative distress and responsibility as an overwhelming impact, leading to profound long-term trauma/distress.

Clinicians expressed high levels of moral distress and burnout while providing care during the pandemic. Burnout due to the conditions of the pandemic was often brought up, impacting family relationships, spousal relationships, and the ability to connect socially with others. Clinicians expressed that pandemic-related changes (e.g., virtual services, accommodating physical distancing, staff redeployment, staff shortages) also led to increased work demands. Additionally, the fear of contracting the COVID-19 virus and increased workloads led to burnout and decreased energy in both their professional and personal lives. The increased workload and no increase in salaries left many clinicians feeling inadequately compensated and underappreciated in their work. Specific challenges with social connection to their colleagues and team cohesion arose when working remotely was another contributor to burnout. Discussion group participants shared that this has also led to an increase in the appeal to increase/shift to private practice because of choice, flexibility, expectations, cost savings and higher compensation.

### Challenges with resources for ED treatment

Youth discussion group participants highlighted the cancellation and delay of services due to the pandemic, longer waitlists, poor access, virtual services including virtual medical monitoring and intake as pandemic-related shifts. In addition, youth and caregivers described challenges accessing ED-informed health professionals, resulting in delays in treatment. When first looking to access ED care, both youth and caregiver participants described challenges with receiving appropriate support from their primary care physicians. One caregiver shared that their family physician admitted to having less than two hours of training on EDs. Youth and families highlighted that stigma around EDs was exacerbated by lack of understanding in primary care, and this stigma and lack of understanding drove intervention delays. In addition to long waitlists and system navigation issues, the strict eligibility criteria and a lack of trained health professionals increased access challenges. Geographically, some youth described accessing care in another province because of a lack of services or access in their region and catchment areas. Many noted that these were challenges already present before the pandemic but were simply amplified during the pandemic.

After being referred for treatment, youth and caregivers further described challenges of being placed on long waiting lists. Cancelled services during the pandemic worsened existing deficits in access to services. Caregivers described a significant decline while on the waitlist for their loved one. While waiting to access services, many caregivers described taking on the responsibility of becoming knowledgeable about how to care for their child. Youth in particular expressed frustration about the lack of prioritization of ED services during the pandemic restrictions.

Clinicians described that there was a general lack of resources during policy and program changes that came in place during the pandemic. For example, clinicians reported the high staff turnover experienced during the pandemic, which was felt at a time when there was an increased demand for services. In addition to the lack of staffing, some clinicians perceived limited bed availability for mental health due to the increase demand for respiratory illnesses.

#### Systemic impacts of delivering or undergoing ED treatment during the pandemic

In addition to the personal costs experienced by participants when delivering or undergoing ED treatment, specific challenges related to the healthcare system during the pandemic were experienced. When discussing overall impacts on ED treatment experiences, many youth and caregivers described high levels of frustration with their experience interacting with untrained health professionals. As a result of over-capacity in limited ED services and other pandemic restrictions affecting capacity (e.g., limited in person programming), youth and caregivers described harmful stigmatizing interactions with medical professionals while waiting in non-specialized ED programs and departments, impacting the quality of care. For example, one caregiver described a nurse who made stigmatizing comments regarding weight and another caregiver described similar comments from a physician. Both situations were described as negatively impacting their child’s recovery.

Although not specific to the pandemic, caregivers described challenges they experienced navigating the system regarding decision-making abilities for their loved one when they are unwell. Several caregivers expanded on the challenges regarding balancing independence for their loved one and the ED with regard to voluntary treatment.

With regards to virtual services, caregivers described some accessibility benefits of virtual care including increased flexibility, transportation savings and less time spent at hospital. Most youth did not describe any benefits with virtual care.

Clinician discussion group participants highlighted that virtual care delivered during the pandemic provided additional support to those living far away from existing services. However, the increase in virtual service offerings and the demand for services also led to an unprecedented increase in workload for clinicians. Specifically, clinicians described challenges with managing the high rates of EDs and maintaining specialized care during the pandemic both in person and virtually. Furthermore, the appeal of private practice specifically during the pandemic drove high staff turnover, resulting in understaffed workplaces which were not well-equipped to meet the demand. The discussion group participants highlighted that the increased demand, along with staff shortages led to feelings of moral distress among health professionals, knowing they were unable to provide care to all those on the waitlist.

## Discussion

The pandemic resulted in an unprecedented rise in rates of EDs among children and youth in Canada propelled by a complex set of circumstances related to the stress of the pandemic, the public health mitigation efforts, and the effects of the lockdowns and restrictions [2]. The current study aimed to explore the costs of the pandemic to those affected by or providing care to youth with EDs and to understand how these times further exacerbated challenges in ED care. Results of this mixed method study revealed that there were a number of direct and indirect medical costs for youth and their families in addition to several personal costs that were absorbed by families with the onset of the pandemic.

Youth and caregivers reported that long waitlists, lack of ED specific treatment, and poor access to qualified mental health staff as the most significant barriers to obtaining ED services during the pandemic. Given the global increase in ED hospitalizations and symptoms during the pandemic [2], these barriers are consistent with the exponential increase in demand for specialized ED services. The majority of youth (75.9%) and caregivers (82.1%) reported that fear of contracting the COVID-19 virus did not affect their decision to engage in ED treatment during the pandemic. No caregivers, and only 6.9% (n = 2) of youth reported the fear of contracting the COVID-19 virus as a barrier to access treatment. Participants described the isolation and anxiety secondary to being in lockdown exacerbated ED symptoms for many youth [2] which outweighed the risks of contracting COVID-19 virus.

Additionally, the pandemic appeared to affect young people, caregivers, and health professionals similarly with the feelings of isolation and loneliness in terms of accessing or delivering services during the pandemic. While public health measures to distance and not socialize were necessary for public safety, it has been questioned for its impact on mental health [1].

Importantly, the top direct medical costs reported by youth and caregivers (i.e., private services, food or nutritional supplements, and medications) are not costs currently captured in existing administrative datasets, making it difficult to quantify the extent of these costs systematically. Greater than 65% of responding youth and caregivers reported accessing private services for treatment of ED symptoms. This is an out-of-pocket cost for youth and families. Long wait times for publicly funded services left many taking on the burden of these costs and/or leaning on private insurance to partially cover costs. However, annual coverage is limited and typically only covers a few sessions [29]. The large proportion of respondents who engaged in services not covered by provincial health care speaks heavily to the lack of publicly funded services for many people living in Canada, and the large gap that exists for specialized ED care. It also points to the high-level of inequity that exists in the system where those who cannot afford services and do not have private insurance are left with limited options for care.

Transportation costs were the top direct nonmedical cost reported by both youth and caregivers. Many respondents reported incurred transportation costs were related to not having ED services near their homes. Thus, incurred costs may be higher for those living in provinces and territories with no ED services or fewer ED services that are dispersed in urban areas. However, virtual care was suggested to have opened up accessibility of services to individuals in rural and remote communities. In total, almost three quarters (Table 2) of health professionals reported they provided virtual care/services during the pandemic. In 2019–2020, 4% of mental health services by physicians were provided virtually compared to 57% in 2020–2021 [30]. Youth and caregivers shared that the transition to virtual care was challenging for medical monitoring and participation. However, a small portion of youth and caregivers (less than 15%) shared there were some cost savings with transportation and accommodations while accessing virtual care. No youth discussion group participants described any benefits of virtual services.

Literature is still evolving regarding cost savings and expenses associated with virtual care. Studied cost savings across Ontario include patient travel time, parking costs, gasoline and public transit costs [31]. Although some respondents shared that virtual care has been successful and cost effective, the evidence continues to emerge regarding virtual care benefits, guidelines and the long-term effectiveness of virtual care in individuals with severe mental illness [32, 33], including youth with EDs. Close to half of youth and caregivers also reported costs associated with accessing virtual care (e.g., computer equipment, internet, etc.). Moving forward from the pandemic, virtual care may offer cost savings to some but increased costs to others. Therefore, further consideration needs to be made to see how flexibility in virtual care can be accessible and provide equitable health care services to complement other ED treatments.

Previous reports have noted the stress and burnout on mental health staff during the pandemic across Canada [34]. Approximately 1 in 3 ED clinicians reported considering leaving or changing their position within the next two years. This is consistent with other healthcare workers in the Statistics Canada survey on *Health Care Worker’s Experiences During the Pandemic* where 34.6% of respondents reported an intention to change jobs in the next three years [35]. Throughout the pandemic, healthcare workers experienced many challenges and changes to their roles. The clinicians in this study reported changes to work dynamics, increased workloads and a growing desire to shift to private practice. The constant cycle of experienced staff moving into private practice is leading to more resources being used to train new inexperienced staff. The challenges with staff resources may continue for the foreseeable future as the demand for ED services continue to remain very high with long waitlists and backlogs [13].

Ongoing discussions are required to determine how these costs can translate into recommendations for systemic change in how ED services are acquired and provided to youth. However, the findings of this study provide some suggestions of how we may reduce different types of costs for youth, families, and health professionals. For example, it is evident that there requires increased training and education of EDs for those working in primary care settings, health professionals outside of specialized ED settings, educators, and others in the community to improve access in the communities where young people live. Increased education and awareness of EDs can also facilitate early detection, appropriate intervention, and combat stigma [36]. Additionally, greater service accessibility financially is recommended. In response to the costs reported related to paying for private services, which are prohibitive for many, subsidizing ED services for youth under provincial health care plans and increasing coverage from private insurance could reduce out of pocket costs to families. Lastly, the paucity of national surveillance ED and costing data infrastructure in our Canadian context to inform policymakers and healthcare leaders of how to address an increase in these types of costs [28] leaves the system ill-equipped to respond properly when needed. There remains a large gap in commitment to research funding for EDs [37] limiting surveillance and costing data opportunities which also undermines these efforts.

## Study strengths and limitations

The strengths of the methodology include a sample consisting of four key groups providing perspective on a variety of community, hospital, and public programs accessed at different stages of the pandemic. The geographical representation in respondents provides national insight into the ongoing challenges and costs associated with ED care across Canada. The mixed methods design allowed for a detailed examination of these costs further strengthening this paper.

Limitations of the present study include a relatively small sample size and a lack of diversity in respondent groups, including limited input from decision-makers. Specifically, it was noted during discussion groups that at least one clinician also identified as a decision-maker, operating in a dual role. Therefore, it could be inferred that other participants may have had a role as a decision maker but chose to participate from the perspective of a clinician. In addition, there were challenges with the anonymized nature of the follow-up engagement process—two caregivers participated twice in the research discussion groups and interviews. For confidentiality reasons and to protect their anonymity of the group, these participants could not have been asked to leave once they were identified as previously participating in a discussion group for this topic. Coders were aware of which discussion groups had duplicate participants and were able to consider this when analyzing the data.

Additional limitations include the extended timeline and potential recall bias of respondents relating to questions that may be related to specific time points during the pandemic. Given the impact and restrictions were in flux throughout the pandemic (beginning March 2020), it may not always be clear which stage of the pandemic or context respondents are referring to in the survey. To mitigate this limitation, within discussion groups, participants were prompted with questions relating to early or later stages of the pandemic where appropriate, to provide additional context to their responses.

Previous work has noted that the pandemic disproportionately affected and exacerbated pre-existing inequities and health disparities among racialized and Indigenous individuals [38–40]. Greater than 85% of respondents in our study self-reported their racial background as white. Given the lack of diversity in respondent’s self-reported race, the experiences reported in this study are not generalizable to racialized individuals. Additionally, most of the youth with lived experience and responding primary caregivers reported a diagnosis of anorexia nervosa, therefore these results may not be generalizable to other ED presentations. Thus, continued efforts should be targeted to recruiting individuals from various racial and minoritized groups and aim to include more representation from individuals with EDs other than anorexia nervosa in order to understand costs among these specific communities.

Given our survey was promoted primarily through ED-related services/networks, this may have contributed to decreased responses from youth and caregivers who have poor access and connection to services, and therefore contributing to an underestimation of costs for those who experience additional barriers to service accessibility. Further, most respondents self-identified as women. Therefore, the reported costs may not accurately reflect the perspective of men, gender-diverse, and non-binary individuals. Geographically, greater than 50% of survey responses were from Ontario and Alberta, which is reflective of the network partners who disseminated the survey and the availability of specialized ED services across Canada, but that limits generalizability of the results to those not represented in those provinces. Despite this, this study serves as a first step in understanding the social and financial costs to youth, caregivers, clinicians, and decision-makers accessing or delivering ED services during the pandemic in Canada.

## Conclusions

This study provided the first examination of the perceived costs incurred by people with EDs living in Canada during the pandemic. It provides an examination of distinct perspectives of the direct and indirect costs generated by people with lived experiences, caregivers and clinicians supporting those with EDs. The study also emphasized the importance of a variety of barriers, including medical, non-medical, access barriers, widespread disparities and inequities, lack of ED specific treatment, and other personal and social costs, to gain knowledge of the multi-factorial burden that impacts those involved in the provision of ED care. It allowed for a close examination of barriers to care, most of which were pre-existing and mainly exacerbated by the pandemic. This data can help inform hospital administrators and policymakers to make decisions regarding future crises and the long-term impact of the pandemic on EDs. This study highlights the toll of the pandemic on those affected by an ED and ignites the conversation about how to be better prepared to best meet the needs of individuals with EDs during crisis and non-crisis times.

### Supplementary Information

**Additional file 1**. Appendix A: Survey Results. Appendix B: Online Survey. Appendix C: Discussion Group Interview Guide

## Data Availability

The authors confirm that the data supporting the findings of this study are available within the article and/or its supplementary materials.

## Abbreviations

ED: Eating disorder
REDCap: Research electronic data capture
