# Supplementary material for: The financial and social impacts of the COVID-19 pandemic on youth with eating disorders, their families, clinicians and the mental health system: a mixed methods cost analysis
Source: J Eat Disord. 2024 Mar 29;12:43. doi: 10.1186/s40337-024-00986-1 (PMC10979568; doi:10.1186/s40337-024-00986-1)
Supplement: Supplementary file 1 — Additional file 1. Appendix A: Survey Results. Appendix B: Online Survey. Appendix C: Discussion Group Interview Guide [file 40337_2024_986_MOESM1_ESM.docx]

**Supplementary Materials**

**Appendix A: Survey Results**

Table 2.1: Youth and primary caregivers’ responses regarding types of costs and barriers to service

|  | Youth  n=29 | Primary Caregivers  n=29 | χ^2a^ |
| --- | --- | --- | --- |
| Direct medical costs (n, %)^1^ |  |  |  |
| Medication | 13 (44.8) | 18 (62.1) | 1.73 |
| Medical Supplies | 1 (3.4) | 6 (20.7) | 4.06 |
| Special Food or Nutritional Supplements | 9 (31.0) | 24 (82.8) | 15.19* |
| Nursing Services | 0 (0.0) | 1 (3.4) | 1.02 |
| Child and Youth Counsellor Services | 3 (10.3) | 7 (24.1) | 1.93 |
| Dietitian Visits | 11 (37.9) | 7 (24.1) | 1.29 |
| Therapist/Psychologist/ Psychiatrist services | 17(58.6) | 17 (58.6) | N/A |
| Diagnostic Imaging | 3 (10.3) | 0 (0.0) | 3.16 |
| Out of Province Services | 1 (3.4) | 1 (3.4) | N/A |
| Out of Country Services | 0 (0.0) | 0 (0.0) | N/A |
| Other health professionals | 5 (17.2) | 4 (13.8) | 0.13 |
| Does not apply | 3 (10.3) | 0 (0.0) | 3.16 |
| Prefer not to answer | 0 (0.0) | 0 (0.0) | N/A |
| Other | 2 (6.9) | 1 (3.4) | 0.35 |
| Direct non-medical costs (n, %)^2^ |  |  |  |
| Transportation | 21 (72.4) | 24 (82.8) | 0.89 |
| Overnight accommodations | 4 (13.8) | 8 (27.6) | 1.68 |
| Childcare for other children while being away | 3 (10.3) | 4 (13.8) | 0.16 |
| Additional supports for primary caregiver | - | 17 (58.6) | - |
| Additional supports for other family members | 9 (31.0) | 11 (37.9) | 0.31 |
| Costs associated with virtual care | 13 (44.8) | 10 (34.9) | 0.65 |
| Other therapies (equine, art) | 3 (10.3) | 5 (17.9) | 0.58 |
| Does not apply | 1 (3.4) | 0 (0.0) | 1.02 |
| Prefer not to answer | 0 (0.0) | 0 (0.0) | N/A |
| Other | 0 (0.0) | 1 (3.4) | 1.02 |
| Indirect costs (n, %)^3^ |  |  |  |
| Unable to attend work resulting in decreased work productivity | 11 (37.9) | 20 (69.0) | 5.61 |
| Unable to attend work resulting in decreased pay | 9 (31.0) | 11 (37.9) | 0.31 |
| Unable to attend work resulting in termination of employment | 3 (10.3) | 2 (6.9) | 0.22 |
| Unable to attend school | 16 (55.2) | 9 (31.0) | 3.45 |
| Tutors/Educational aids to support missed schoolwork | 8 (27.6) | 2 (6.9) | 4.35 |
| Loss of extended family support due to COVID-19 restrictions | 7 (24.1) | 10 (34.5) | 0.75 |
| Increased feelings of isolation due to COVID-19 policies | 14 (48.3) | 17 (58.6) | 0.62 |
| Does not apply | 5 (17.2) | 0 (0.0) | 5.47 |
| Prefer not to answer | 0 (0.0) | 0 (0.0) | N/A |
| Other | 0 (0.0) | 0 (0.0) | N/A |
| Cost savings (n, %)^4^ |  |  |  |
| Parking at discounted rates | 1 (3.4) | 3 (10.3) | 1.07 |
| Food vouchers | 2 (6.9) | 1 (3.4) | 0.35 |
| Savings due to virtual care | 3 (10.3) | 4 (13.8) | 0.16 |
| Federal or Provincial level benefits | 6 (20.7) | 5 (17.2) | 0.11 |
| Did not experience any cost savings or financial aid | 16 (55.2) | 12 (41.4) | 1.10 |
| Other | 1 (3.4) | 0 (0.0) | 1.02 |
| Barriers to services/treatment (n, %)^5^ |  |  |  |
| Difficulty accessing technology for virtual care | 4 (13.8) | 5 (17.2) | 0.13 |
| Obstacles in transitioning groups online | 7 (24.1) | 2 (6.9) | 3.29 |
| Costs of services/treatment | 15 (51.7) | 8 (27.6) | 3.53 |
| Family responsibilities | 8 (27.6) | 7 (24.1) | 0.09 |
| Unable to find therapist/treatment | 11 (37.9) | 14 (48.3) | 0.63 |
| Long waiting lists | 17 (58.6) | 21 (72.4) | 1.22 |
| Lack of eating-disorder specific treatment | 17 (58.6) | 18 (62.1) | 0.07 |
| Lack of access to qualified mental health professionals | 8 (27.6) | 20 (69.0) | 9.94* |
| Mental health staffing shortages | 14 (48.3) | 13 (44.8) | 0.07 |
| Interruption of service | 12 (41.4) | 6 (20.7) | 2.90 |
| Coronophobia (fear of contracting the virus causing COVID-19) | 2 (6.9) | 0 (0.0) | 2.07 |
| Does not apply- did not engage in services during the COVID-19 pandemic | 0 (0.0) | 1 (3.4) | 1.02 |
| Does not apply- there were no barriers to accessing services during the COVID-19 pandemic | 1 (3.4) | 2 (6.9) | 0.35 |
| Other | 2 (6.9) | 0 (0.0) | 2.07 |

^a^df=1

^1^Direct medical costs Bonferroni corrected for χ^2^, *p<0.0038

^2^Direct non-medical costs Bonferroni corrected for χ^2^, *p<0.0055

^3^Indirect costs Bonferroni corrected for χ^2^, *p<0.00625

^4^Cost savings Bonferroni corrected for χ^2^, *p<0.0083

^5^Barriers to services/treatment Bonferroni corrected for χ^2^, *p<0.0038

Table 2.2: Clinician and decision-maker responses regarding shifts in work and experienced costs

|  | Health professionals (n=56^1^) |
| --- | --- |
| Change in Work (n, %) |  |
| Redeployed to other services | 7 (12.5) |
| Remote work/working from home | 29 (51.8) |
| Provide virtual care/services | 39 (69.6) |
| Change in work hours | 13 (23.2) |
| Other | 8 (14.3) |
| Personal costs or impacts (n, %) |  |
| Fear/isolation due to COVID-19 exposure at workplace | 33 (58.9) |
| Increased childcare expenses due to change in work hours | 6 (11.5) |
| Technology expenses related to working remotely | 23 (41.1) |
| Increased work expectations/demands | 36 (64.3) |
| Decreased productivity | 18 (32.1) |
| Other | 10 (17.9) |
| Cost savings (n, %) |  |
| One-time pandemic bonus pay | 21 (37.5) |
| Temporary pandemic bonus on hourly rate | 6 (10.7) |
| Discounted parking rates | 16 (28.6) |
| Food vouchers | 2 (3.6) |
| Childcare cost savings due to remote work | 2 (3.6) |
| Cost savings due to remote work (e.g., transportation...etc.) | 15 (26.8) |
| Increased work productivity | 7 (12.5) |
| Other | 3 (5.4) |

^1^includes 52 clinicians and 4 decision makers

**Appendix B: Online Survey**

**Question for all respondents**

Please indicate the perspective from which you are responding to this survey

- between 16-25 years old who currently lives with or has lived with an eating disorder and/or disordered eating
- a primary caregiver of a child or youth (up to 25 years old) with an eating disorder and/or disordered eating
- a clinician or health professional who works with children or youth (up to 25 years old) with eating disorders and/or disordered eating
- a decision-maker and/or administrator working in health-related or community-based organizations in the field of eating disorders for children or youth (up to 25 years old)

*A primary caregiver is the person who, over the past 12 months, has spent the most time helping an individual with an eating disorder and/or disordered eating*

**Young Person**

About Diagnosis

1. Which eating disorder were you primarily diagnosed with? Please check all that apply.

- Diagnosed with Anorexia Nervosa
- Diagnosed with Bulimia Nervosa
- Diagnosed with Binge Eating Disorder
- Diagnosed with Avoidant Restrictive Food Intake Disorder
- Diagnosed with Eating Disorder Not Otherwise Specified (EDNOS)
- Other Specified Feeding and Eating Disorders (OSFED), including Atypical Anorexia Nervosa
- Unspecified Feeding or eating disorder (UFED)
- I have not had a formal diagnosis; however, I experience symptoms of an eating disorder/disordered eating
- Do not know
- Prefer not to answer
- Other not listed above
  - - Other. Please specify:

1. What was your age in years when you first began experiencing eating disorder symptoms?
2. How long after symptoms began did you engage in services (e.g., doctor, dietician) regarding your eating disorder symptoms?
   - Less than three months after symptoms began
   - Greater than 3 months but less than 6 months after symptoms began
   - Greater than 6 months but less than a year after symptoms began
   - Greater than a year but less than 3 years after symptoms began
   - Longer than 3 years after symptoms began
   - Do not know
   - I have not yet sought services regarding eating disorder symptoms
3. How long after symptoms began were you formally diagnosed with an eating disorder?

- Less than three months after symptoms began
- Greater than 3 months but less than 6 months after symptoms began
- Greater than 6 months but less than a year after symptoms began
- Greater than a year but less than 3 years after symptoms began
- Longer than 3 years after symptoms began
- Do not know
- I have not had a formal diagnosis; however, I experience symptoms of an eating disorder and/or disordered eating

1. Currently, are you experiencing eating disorder symptoms?
   1. I am in recovery
      1. What was your age in years when you no longer experienced eating disorder symptoms and began recovery?
   2. I am experiencing eating disorder symptoms
   3. Other
      1. Please comment
2. What services/treatment have you engaged in for treatment of eating disorder symptoms? Please check all that apply.

- Intensive hospital-based services (publicly funded)
- Outpatient hospital-based services (publicly funded)
- Outpatient community-based services (publicly funded)
- Community services or supports (offered free or on a sliding scale)
- Private services (e.g. paid treatment services with a psychologist, social worker, dietician, carer skills workshops, etc)
- Treatment or monitoring with a health provider(s) (e.g. a family physician, pediatrician, nurse practitioner, primary care network, family health team, etc)
- I have not yet engaged in any eating disorder services/treatment
- Other Services/Treatment
  - Please specify:

Covid-19

1. Did you receive services/treatment for eating disorder symptoms **before** the COVID-19 pandemic (before March 2020)?
   - Yes
   - No
2. Were the eating disorder services/treatment you were accessing interrupted or put on hold after the pandemic began (March 2020)?

- Yes - Services/treatment were interrupted or put on hold after the pandemic began
  - Please briefly describe how eating disorder services/treatment were interrupted after the pandemic began (delays, cancellations...etc.)
  - No - Services/treatment continued with no interruptions after the pandemic began
- Does not apply - I was not receiving services when the pandemic began
- Do not know

1. Did you seek/receive eating disorder services/treatment **during** the COVID-19 pandemic (March 2020 to present)?
   - Yes
   - No
2. Did fear of contracting COVID-19 affect your decision to engage in eating disorder services/treatment during the pandemic (March 2020 to present)?

- Yes
  - If yes, please explain
- No
- Do not know
- Does not apply – did not require eating disorder services/treatment during the COVID-19 pandemic.

1. Which of the following were **barriers to accessing** services/treatment during the COVID-19 pandemic? Please check all that apply.

- Difficulty accessing technology for virtual care
- Obstacles in transitioning groups online
- Cost of services/treatment
- Family responsibilities
- Unable to find therapist/treatment
- Long waiting lists
- Lack of eating disorder specific treatment
- Lack of access to qualified mental health professionals
- Mental health staffing shortages
- Interruption of service
- Coronophobia (fear of contracting the virus causing COVID-19)
- Does not apply - did not engage in services during the COVID-19 pandemic
- Does not apply - there were no barriers to accessing services during the COVID-19 pandemic
- Other
  - Other barriers. Please specify:

Costs

The next questions ask about the types of costs you or your family may have had while receiving treatment/services for your eating disorder during the COVID-19 pandemic

**If no to receiving treatment during pandemic: You have indicated that you did not require or engage in eating disorder treatment/services during the COVID-19 pandemic (March 2020 until present), therefore no further questions regarding eating disorder services/treatment will be asked. Please continue to the last section “About you”.**

1. At the time you were receiving treatment/services, were you (and/or your family) ever responsible for covering part or/all your own costs?
   - Yes
   - No
   - Don’t know
2. Which of the following **direct medical costs** did you/your family experience (**not covered by provincial healthcare**) while accessing services/treatment for your eating disorder during the COVID-19 pandemic? Please check all that apply.

- Medication (Prescription drugs, over the counter medications...etc.)
- Medical Supplies
- Special Food or Nutritional Supplements
- Nursing Services
- Child and Youth Counsellor Services
- Dietitian Visits
- Therapist/Psychologist/ Psychiatrist services
- Diagnostic Imaging
- Out of Province Services
- Out of Country Services
- Other health professionals for other health consequences as a result of the eating disorder (e.g. dental services...etc.)
- Does not apply
- Prefer not to answer
- Other
  - Other direct medical costs. Please specify:

Note: Direct medical costs can include the costs of medication, therapy. Etc.

1. **Only for those who say yes to being responsible for their own costs:** On average, what percentage of these **direct medical costs** were you (and/or your family) paying out of pocket (not covered by private insurance)?

[responses put on a sliding scale 0-100 %, Zero being “none” and 100 being “paying all costs out of pocket”]

1. Which of the following **direct non-medical costs** did you/your family experience (not covered by provincial healthcare) while accessing services/treatment for your eating disorder during the COVID-19 pandemic? Please check all that apply.

- Transportation (bus fare, gas, parking...etc.)
- Overnight accommodations
- Childcare for other children while being away
- Additional supports (e.g, family therapy, group counselling.) for other family members
- Costs associated with virtual care (e.g, computer equipment, internet ….etc).
- Other therapies (e.g, equine, art therapy…etc.)
- Does not apply
- Prefer not to answer
- Other
  - Other direct non-medical costs. Please specify:

Note: Direct non-medical costs are expenditures as the result of an illness but are not involved in the direct purchasing of medical services

1. **Only for those who say yes to being responsible for their own costs:** On average, what percentage of these **direct non-medical** **costs** were you (and/or your family) paying out of pocket (not covered by private insurance or provincial healthcare)?

[responses put on a sliding scale 0-100 %, Zero being “none” and 100 being “paying all costs out of pocket”]

1. Which of the following **indirect costs** did you/your family experience (not covered by provincial healthcare) while accessing services/treatment for your eating disorder during the COVID-19 pandemic? Please check all that apply.
   - Unable to attend work resulting in decreased work productivity
   - Unable to attend work resulting in decreased pay
   - Unable to attend work resulting in termination of employment
   - Unable to attend school
   - Tutors/Educational aids to support missed schoolwork
   - Loss of extended family support due to COVID-19 restrictions
   - Increased feelings of isolation due to COVID-19 policies (e.g. one caregiver at a time)
   - Does not apply
   - Prefer not to answer
   - Other
     - - Other indirect costs. Please specify:

Note: Indirect costs are the lost earnings and productivity by the patient or caregivers related to the illness.

1. **Only for those who say yes to being responsible for their own costs:** On average, what percentage of these **indirect costs** were you (and/or your family) paying out of pocket (not covered by private insurance or provincial healthcare)?

[responses put on a sliding scale 0-100 %, Zero being “none” and 100 being “paying all costs out of pocket”]

1. Which of the following **cost savings or financial aid** did you/your family receive while accessing eating disorder treatment/services? Please check all that apply.

- Parking at discounted rates
- Food vouchers
- Savings due to virtual care
  - Please explain how **virtual care** resulted in cost savings for you/your family
- Federal or Provincial level benefits/ tax credits because of the eating disorder (e.g, Child disability benefit)
- Did not experience any cost savings or financial aid
- Other

1. Please share any **additional comments** you have regarding the costs associated with accessing eating disorder services/treatment during the COVID-19 pandemic.

Youth Respondents would then complete “about you section” (pg.17)

**Primary Caregiver**

1. What is the best way to describe your relationship to the individual with an eating disorder, whom you are/were a primary caregiver for? If none of these options describe your relationship, please select "your loved one".
   - Your child
   - Your sibling
   - Your foster child
   - Your grandchild
   - Your partner/spouse
   - Your loved one

About Diagnosis

*Note all blanks will be replaced by the described relationship indicated in the question above*

1. Which eating disorder was_________ primarily diagnosed with? Please check all that apply.

- Diagnosed with Anorexia Nervosa
- Diagnosed with Bulimia Nervosa
- Diagnosed with Binge Eating Disorder
- Diagnosed with Avoidant Restrictive Food Intake Disorder
- Diagnosed with Eating Disorder Not Otherwise Specified (EDNOS)
- Other Specified Feeding and Eating Disorders (OSFED), including Atypical Anorexia Nervosa
- Unspecified Feeding or eating disorder (UFED)
- They have not had a formal diagnosis; however, they experience symptoms of an eating disorder and/or disordered eating
- Do not know
- Prefer not to answer
- Other not listed above
  - Other. Please specify:

1. What was ____________’s age in years when they first began experiencing eating disorder symptoms?
2. What is ______________’s current age in years? [open text box]
3. Please select the response or responses that best describe your ______________’s gender identity:

- Woman
- Man
- Non-Binary
- Gender fluid
- Two-spirit
- Another gender identity
  - - Another gender identity. Please specify:
- I prefer not to respond

1. How long after symptoms began did ___________ engage in services (e.g. doctor, dietician) regarding eating disorder symptoms?

- Less than three months after symptoms began
- Greater than 3 months but less than 6 months after symptoms began
- Greater than 6 months but less than a year after symptoms began
- Greater than a year but less than 3 years after symptoms began
- Longer than 3 years after symptoms began
- Do not know
- They have not yet sought services regarding eating disorder symptoms

1. How long after symptoms began was ___________ formally diagnosed with an eating disorder?

- Less than three months after symptoms began
- Greater than 3 months but less than 6 months after symptoms began
- Greater than 6 months but less than a year after symptoms began
- Greater than a year but less than 3 years after symptoms began
- Longer than 3 years after symptoms began
- Do not know
- They have not had a formal diagnosis; however, they experience symptoms of an eating disorder/disordered eating

1. Currently, is ___________ experiencing eating disorder symptoms?

- In recovery
  - How old in years was ______ when they no longer experienced eating disorder symptoms and started recovery?
- Currently experiencing symptoms
- Other
  - Please comment

1. What services/treatment have you/ ______________engaged in for treatment of their eating disorder symptoms? Please check all that apply.

- Intensive hospital-based services (publicly funded)
- Outpatient hospital-based services (publicly funded)
- Outpatient community-based services (publicly funded)
- Community services or supports (offered free or on a sliding scale)
- Private services (e.g. paid treatment services with a psychologist, social worker, dietician, carer skills workshops, etc)
- Treatment or monitoring with a health provider(s) (e.g. a family physician, pediatrician, nurse practitioner, primary care network, family health team, etc)
- They have not yet received eating disorder services/treatment
- Other Services/Treatment
  - Please specify

Covid-19

1. Did ______________ receive services/treatment for eating disorder symptoms **before** the COVID-19 pandemic (before March 2020)?
   - Yes
   - No
2. Were the eating disorder services/treatment ________ was accessing interrupted or put on hold after the pandemic began (March 2020)?

- Yes - Services/treatment were interrupted or put on hold after the pandemic began
  - Please briefly describe how eating disorder services/treatment were interrupted after the pandemic began (delays, cancellations...etc.)
  - No - Services/treatment continued with no interruptions after the pandemic began
- Does not apply - they were not receiving services when the pandemic began
- Do not know

1. Did ___________ receive eating disorder services/treatment **during** the COVID-19 pandemic (March 2020 to present)?
   - Yes
   - No
2. Did fear of contracting COVID-19 affect ____________ decision to engage in eating disorder services/treatment during the pandemic (March 2020 to present)?

- Yes
  - If yes, please explain
- No
- Do not know
- Does not apply – did not require eating disorder services treatment during the COVID-19 pandemic.

1. Which of the following were **barriers to accessing** services/treatment during the COVID-19 pandemic?
   - Difficulty accessing technology for virtual care
   - Obstacles in transitioning groups online
   - Cost of services/treatment
   - Family responsibilities
   - Unable to find therapist/treatment
   - Long waiting lists
   - Lack of eating disorder specific treatment
   - Lack of access to qualified mental health professionals
   - Mental health staffing shortages
   - Interruption of service
   - Coronophobia (fear of contracting the virus causing COVID-19)
   - Does not apply - did not engage in services during the COVID-19 pandemic
   - Does not apply - there were no barriers to accessing services during the COVID-19 pandemic
   - Other
     1. Other barriers. Please specify:

Costs

The next questions ask about the types of costs you or your family incurred while ______ received eating disorder treatment/services during the COVID-19 pandemic.

**If no to receiving treatment during pandemic: You have indicated that _____________ did not require or engage in eating disorder treatment/services during the COVID-19 pandemic (March 2020 until present), therefore no further questions regarding eating disorder services/treatment will be asked. Please continue to the last section “About you”.**

1. Which of the following **direct medical costs** did you/your family incur (not covered by provincial healthcare) because of _________'s eating disorder during the COVID-19 pandemic? Please check all that apply.

- Medication (Prescription drugs, over the counter medications...etc.)
- Medical Supplies
- Special Food or Nutritional Supplements
- Nursing Services
- Child and Youth Counsellor Services
- Dietitian Visits
- Therapist/Psychologist/ Psychiatrist services
- Diagnostic Imaging
- Out of Province Services
- Out of Country Services
- Other health professionals for other health consequences as a result of the eating disorder (e.g. dental services...etc.)
- Does not apply
- Prefer not to answer
- Other
  - Other direct medical costs. Please specify:

1. On average, what percentage of these **direct medical costs** were you paying out of pocket (not covered by private insurance)?

[responses put on a sliding scale 0-100 %, Zero being “none” and 100 being “paying all costs out of pocket”]

1. Which of the following **direct non-medical costs** did you/your family incur (not covered by provincial healthcare) because of ________'s eating disorder during the COVID-19 pandemic? Please check all that apply.
   - Transportation (bus fare, gas, parking...etc.)
   - Overnight accommodations
   - Childcare for other children while being away
   - Additional supports for myself as a primary caregiver (e.g, coping mechanism, therapy…etc._
   - Additional supports for other family members (e.g, therapy, group counselling)
   - Costs associated with virtual care (e.g, computer equipment, internet ….etc.
   - Other therapies (e.g, equine, art therapy…etc.
   - Does not apply
   - Prefer not to answer
   - Other
     - - Other direct non-medical costs. Please specify:

Note: Direct non-medical costs are expenditures as the result of an illness but are not involved in the direct purchasing of medical services

1. On average, what percentage of these **direct non-medical** **costs** were you paying out of pocket (not covered by private insurance or provincial healthcare)?

[responses put on a sliding scale 0-100 %, Zero being “none” and 100 being “paying all costs out of pocket”]

1. Which of the following **indirect costs** did you/your family incur (not covered by provincial healthcare) because of __________'s eating disorder during the COVID-19 pandemic? Please check all that apply.
   - Unable to attend work resulting in decreased work productivity
   - Unable to attend work resulting in decreased pay
   - Unable to attend work resulting in termination of employment
   - Unable to attend school
   - Tutors/Educational aids to support missed schoolwork
   - Loss of extended family support due to COVID-19 restrictions
   - Increased feelings of isolation due to COVID-19 policies (e.g. one caregiver at a time)
   - Does not apply
   - Prefer not to answer
   - Other
     1. Other indirect costs. Please specify:

Note: Indirect costs are the lost earnings and productivity by the patient or caregivers related to the illness.

1. On average, what percentage of these **indirect costs** were you paying out of pocket (not covered by private insurance or provincial healthcare)?

[responses put on a sliding scale 0-100 %, Zero being “none” and 100 being “paying all costs out of pocket”]

1. Which of the following **savings or financial aid** did you/your family receive because of COVID-19 policies while accessing eating disorder treatment/services for __________? Please check all that apply.

- Parking at discounted rates
- Food vouchers
- Federal or Provincial level benefits/ tax credits because of the eating disorder (e.g, Child disability benefit)
- Savings due to virtual care
  - Please explain how virtual care resulted in **cost savings** for you/your family
- Did not experience any cost savings or financial aid
- Other
  - 1. Other cost savings. Please specify:

1. Please share any **additional comments** you have regarding the costs associated with accessing eating disorder services/treatment for _________during the COVID-19 pandemic.

Primary Caregivers would then complete “about you section” (pg.17)

**Clinicians**

1. How long have you been working in the field of eating disorders?

- Less than six months
- Greater than six months but less than a year
- Greater than a year but less than five years
- Greater than five years but less than ten years
- Greater than ten years

1. Have you been working as a clinician in the field of eating disorders for the entire duration of the  **COVID-19 pandemic** (March 2020 to present) ?

- Yes
- No
  - 1. Please indicate how many months you have been working as a clinician in the field of eating disorders during the COVID-19 pandemic (March 2020 to present) (INSERT integer)

1. Which of the following ways did your practice shift during the COVID-19 pandemic? Please check all that apply.

- Redeployed to other services
- Remote work/working from home
- Provide virtual care/services
- Change in work hours
- Other
  - 1. Other please specify

1. Please elaborate on how your practice has shifted during the COVID-19 pandemic.

Costs

1. Which of the following were **personal costs or impacts** you experienced while working during the COVID-19 pandemic (March 2020 to present)? Please check all that apply.

- Fear/isolation due to COVID-19 exposure at workplace
- Increased childcare expenses due to change in work hours
- Technology (Internet, equipment...etc.) expenses related to working remotely
- Increased work expectations/demands
- Decreased productivity
- Other
  - Other. Please specify:

1. Which of the following **cost savings**, if any, did you experience because of working during the COIVD-19 pandemic? Please check all that apply.

- One-time pandemic bonus pay
- Temporary pandemic bonus on hourly rate
- Discounted parking rates
- Food vouchers
- Childcare cost savings due to remote work
- Cost savings due to remote work (e.g, transportation...etc.)
- Increased work productivity
- Other
  - Other. Please specify:

1. Are you considering changing or leaving your current position in the next 1-2 years?
   1. Yes
      1. Has working during the COVID-19 pandemic impacted this decision?
         1. Yes, please explain.
   2. No
2. Please share any **additional comments**you have on how the COVID-19 pandemic has had on **costs** to you as an individual and/or your practice.

Clinicians would then complete “about you section” (pg.17)

**Decision Makers/ Administrators**

1. How long have you been working in the field of eating disorders?
   - Less than 6 months
   - Greater than six months but less than a year
   - Greater than a year but less than five years
   - Greater than five years but less than ten years
   - Greater than 10 years
2. Have you been working in a decision-making capacity in the field of eating disorders for the entire duration of the  **COVID-19 pandemic** (March 2020 to present) ?
   - Yes
   - No
     1. Please indicate how many months you have been working as a decision-maker in the field of eating disorders during the COVID-19 pandemic (March 2020 to present) (INSERT integer)
3. Which of the following ways did your practice shift during the COVID-19 pandemic? Please check all that apply.
   - Redeployed to other services
   - Complete remote work
   - Provide virtual care/services
   - Change in work hours
   - Other
     1. Other. Please specify:
4. Please elaborate on how your administrative duties/organization obligations have shifted during the COVID-19 pandemic.

Costs

1. Which of the following were **personal costs or impacts** you experienced while working during the COVID-19 pandemic (March 2020 to present)? Please check all that apply.

- Fear/isolation due to COVID-19 exposure at workplace
- Increased childcare expenses due to change in work hours
- Technology (Internet, equipment...etc.) expenses related to working remotely
- Increased work expectations/demands
- Decreased productivity
- Other
  - Other. Please specify:

1. Which of the following **cost savings**, if any, did you experience because of working during the COIVD-19 pandemic? Please check all that apply.

- One-time pandemic bonus pay
- Temporary pandemic bonus on hourly rate
- Discounted parking rates
- Food vouchers
- Childcare cost savings due to remote work
- Cost savings due to remote work (e.g, transportation...etc.)
- Increased work productivity
- Other
  - Other. Please specify:

1. Please describe any **challenges/opportunities** experienced while responding to restrictions and policies during **early stages of the pandemic (first wave).**
2. Please describe any **challenges/opportunities** you currently or did experience while responding to restrictions and policies during **other stages** of the pandemic.
3. Are you considering changing or leaving your current position in the next 1-2 years?

- Yes
  - 1. Has working during the COVID-19 pandemic impacted this decision?
       1. Yes, please explain.
- No

1. Please share any **additional comments**you have on how the COVID-19 pandemic has had on **costs** to you as an individual and/or your organization.

Decision-makers/administrators would then complete “about you section” (pg.17)

**About You**

This next group of questions is intended to help us know the range of individuals who have completed the survey.

NOTE: for primary caregivers: We have collected sufficient information regarding __________. Please respond to the next series of questions about **yourself** as the primary caregiver for your ____________

1. How old are you?
2. We are inviting people from all over Canada to participate. Please enter the first three digits of your postal code
3. Please select the response or responses that best describe your gender identity:

- Two-spirit
- Gender diverse
- Man
- Non-Binary
- Woman
- Another gender identity [Text Box]
- I prefer not to respond

1. Do you identify as transgender?

- Yes
- No
- I prefer not to respond

1. Please select the response or responses that best describe your sexual orientation:

- Two-spirit
- Aromantic
- Asexual
- Bisexual
- Gay
- Heterosexual (straight)
- Lesbian
- Pansexual
- Queer
- Questioning
- Another Orientation
- I prefer not to respond

1. Do you self-identify as an Indigenous person of North America (First Nations, Metis, Inuit, Native American)?

- Yes, First Nations
- Yes, Inuk/Inuit
- Yes, Metis
- No
- Do not know
- Prefer not to answer
- Other

1. If you identify yourself as a First Nations person, do you live on a reserve or off-reserve. This information is collected because eating disorder services may be different on or off a reserve.

- Off-reserve
- On a reserve
- Prefer not to answer

1. In our society, people are often described by their race or racial backgroung. These are not based in science, but racism may affect the way we are treated by others and this can affect our health. Which category(ies) best describes you? Please note the examples do not cover all races or racial backgrounds. Check all that apply.

- Indigenous (Such as First Nations, Inuk/Inuit, Métis descent)
- Black (Such as African Diaspora, Caribbean, Black Canadian, etc.)
- Central/North/West Asian (Such as Afghan, Persian, Egyptian, Iranian, Kurdish, Lebanese, Turkish, etc.)
- East Asian (Such as Chinese, Japanese, Korean, etc.)
- Latin X or Latin American (Such as South American, Caribbean, Mesoamerican, etc.)
- South Asian (Such as Bangladeshi, Indian, Pakistani, Sri Lankan, etc.)
- Southeast Asian (Such as Cambodian, Filipino, Indonesian, Thai, Vietnamese, etc.)
- White (Such as European, French, British, etc.)
- Another race category
  - TEXTBOX
- Do not know
- Prefer not to answer

1. Do you or your family currently experience financial difficulties (i.e. paying for housing, food, transportation, school supplies)

- Yes
- No
- Not sure
- I prefer not to respond

1. Do you live with one or more disabilities?
   1. Yes
      1. Please indicate the type of long-term or reoccruing condition(s)/disabilites that you experience. Please check all that apply. Note that these examples do not cover all conditions.
         - Addictions (such as alcohol, recreational or prescription drugs, gaming, etc.)
      - Blind, partial or vision loss
      - Chronic and episodic conditions (such as Diabetes, cancer, epilepsy, etc.
      - Deafness, partial or complete hearing loss
      - Environmental sensitivities (such as sensitivity to lights, sounds, etc..)
      - Intellectual or neurological disabilities (such as Autism spectrum, traumatic brain injuries, Attention Deficit 7, Hyperactivity Disorder, etc.)
      - Learning disabilities (such as Dyslexia, etc.)
      - Mobility limitations (such as difficulty moving, uses a wheelchair, walker, etc.)
      - Speech impairment (such as non-verbal, etc.)
      - Other
        - TEXTBOX

- Prefer not to answer

**Appendix C - Discussion Group Interview Guide**

**Goals**

- Explore areas that are over/underrepresented in the survey responses
- Share/obtain feedback on major themes generated from the survey with the idea of working to identify key messaging that needs to be shared by policymakers and government
- Identify the potential differences for specific communities/provinces in their needs. (What to be considered for different communities)?

**Questions**

1. Opening question

- Youth: Did you access eating disorder services during the pandemic? Was this the first time you received services for your ED?
- Primary Caregivers: Did your loved one access eating disorder services during the pandemic? Was this the first time your loved one received services for your ED?
- Clinicians/Decision-makers: What is your role and how long have you been working in the field of eating disorders or mental health?

1. The following are a list of themes identified by [young people/primary caregivers/clinicians/decision-makers] about the costs of eating disorder treatment/services during the pandemic. [List 3-5 key themes from survey on a powerpoint slide and put themes in chat]

- Do these align with your experience in your local community and what else would you add?
- Are any of these themes surprising, what you would have expected?
- Do you have any specific examples of how your experience relates to one or more of these themes?

1. The following are a list of gaps, missing responses in our survey, do you have any comments on why we have these gaps? [List 3-5 key themes from survey on a powerpoint slide and put themes in chat]

1. Based on your experience what is the main message that we should tell policymakers/decision-makers about how we can improve the system based on your experiences during COVID?

- If there was another pandemic, how would you like to see our system respond?
- Is there anything about your specific community (or communities like yours) that needs to be considered?

1. Is there anything else regarding how the COVID-19 pandemic affected the costs of [accessing/providing] eating disorder services that you would like to share?
